# Comparison of colostrum and milk extracellular vesicles small RNA cargo in water buffalo

**DOI:** 10.1038/s41598-024-67249-6

**Published:** 2024-08-03

**Authors:** Samanta Mecocci, Daniele Pietrucci, Marco Milanesi, Stefano Capomaccio, Luisa Pascucci, Chiara Evangelista, Loredana Basiricò, Umberto Bernabucci, Giovanni Chillemi, Katia Cappelli

**Affiliations:** 1 Department of Veterinary Medicine, University of Perugia, 06126 Perugia, Italy; 2 Department for Innovation in Biological, Agro-Food and Forest Systems (DIBAF), University of Tuscia, 01100 Viterbo, Italy; 3 Department of Agriculture and Forest Sciences (DAFNE), University of Tuscia, 01100 Viterbo, Italy; 4 Institute of Translational Pharmacology, National Research Council, CNR, 00133 Rome, Italy

**Keywords:** Colostrum EVs, Milk EVs, Italian Mediterranean buffalo, Small RNA-seq, Immunomodulatory molecules, miRNAs, Animal biotechnology, RNA sequencing, Epigenetics, Non-coding RNAs

## Abstract

Recently, much interest has been raised for the characterization of signaling molecules carried by extracellular vesicles (EVs), which are particularly enriched in milk (mEVs). Such interest is linked to the capability of EVs to cross biological barriers, resist acidification in the gastric environment, and exert modulation of the immune system, mainly through their microRNA (miRNA) content. We characterized the small-RNA cargo of colostrum EVs (colosEVs) and mEVs from Italian Mediterranean buffalo through next generation sequencing. Colostrum (first milking after birth) and milk (day 50 of lactation) were sampled from seven subjects from five farms. ColosEVs and mEVs were subjected to morphological characterization, followed by high-depth sequencing of small RNA libraries produced from total RNA. The main difference was the amount of EV in the two samples, with colostrum showing 10 to 100-fold higher content than milk. For both matrices, miRNA was the most abundant RNA species (95% for colosEVs and 96% for mEVs) and three lists were identified: colosEV-specific, mEV-specific and shared most expressed. Gene ontology (GO) enrichment analysis on miRNA targets highlighted many terms related to the epigenetic, transcriptional and translational regulations across the three lists, with a higher number of enriched terms for colosEV-specific miRNAs. Terms specific to colosEVs were related to “cell differentiation” and “microvillus assembly”, while for mEV “cardiac and blood vessel development” and “mitochondria” emergerd. Immune modulation terms were found for both sample-specific miRNAs. Overall, both matrices carry a similar molecular message in terms of biological processes potentially modulated into receiving cells, but there is significant difference in the abundance, with colostrum containing much more EVs than milk. Moreover, colosEVs carry molecules involved in signal transduction, cell cycle and immune response, as for mEVs and EVs of other previously characterized species, but with a special enrichment for miRNAs with epigenetic regulation capacities. These beneficial characteristics of colosEVs and mEVs are essential for the calf and could also be exploited for the therapeutic purposes in humans, although further studies are necessary to measure the sanitization treatment impact on EV conservation, especially in buffalo where milk is consumed almost exclusively after processing.

## Introduction

Milk constitutes the main and mostly unique source of nutritional elements for mammalian newborns, and even more, a complex delivery system for maternal messages toward the offspring^1^.

The presence of bioactive compounds in milk makes it an important source of nutrients for human consumers as well^2^. Milk is richer in all major constituents, with high energy and nutritional value conferred by fat, which is one of the main fractions^3,4^. Moreover, buffalo milk offers advantages over milk from other species in promoting health due to specific properties such as a higher protein content, and calcium, the main mineral, 1.5-fold higher than in cow milk^3^. Furthermore, milk from buffalo is characterized by a high content of antioxidant and anti-inflammatory molecules such as tocopherols and vitamin A^2,4^, and contains exclusive compounds including biliverdin, bioactive pentasaccharides, and gangliosides^5^. The microbial composition of buffalo milk has also been evaluated in detail, identifying characteristics that qualify it as a functional food with probiotic effects^6^. As expected, the milk composition of these bioactive molecules can change over time, in relation to the lactation period, environmental stimuli, diet, and genetic factors^7–10^.

Colostrum shows a significant different composition compared to milk^11^; for buffaloes, as for all other ruminant species, colostrum represents a crucial source of immunoglobulins for newborns since the syndesmochorial placenta inhibits the transfer of antibodies. This aspect makes buffalo newborns particularly susceptible to infections, and it is the main reason for the importance of colostrum intake^12^. In addition to the higher protein content compared with mature milk, colostrum also contains greater levels of total solids, non-fat solids, and milk urea nitrogen, while exhibiting lower levels of fat and lactose^13^. For minerals, buffalo colostrum has high concentrations of Na, Mg, Co, Fe, and K, while milk is more concentrated in Ca^13^.

Despite the importance of buffalo milk for dairy products, few studies have been conducted to evaluate its colostrum composition, and only one evaluated buffalo milk extracellular vesicles (mEVs)^14^. Extracellular vesicles (EVs) are spherical micro/nano-sized cell structures^15,16^ characterized by a phospholipid bilayer enclosing a plethora of molecules, including RNAs, lipids, proteins and DNA fragments^17–20^. Basing on recent developed guidelines of the international society for extracellular vesicles (ISEV)^21^ and the increasing knowledge about these particular structures, EVs are mainly subdivided into three categories with respect to the size: small (30–150 nm), medium-large (100–1000 nm) and apoptotic bodies (50–5000 nm). Small and medium-large EVs can refer to two main vesicle typologies, exosome and microvesicles, which differ in the biogenesis process^22^. Since no specific molecular markers exist to distinguish between these two subtypes and the co-isolation quite always occurs, it is desirable to use the nomenclature based on size (small/medium-large EVs).

EVs can be released by all cell types in the extracellular matrix and can reach close and distant receiving cells, which, after the vesicle absorption, can undergo the modulation of cellular processes^23^. Indeed, EV uptake can induce a modification in the recipient cell toward an anti-inflammatory, anti-cancer, pro-regenerative or angiogenetic phenotype. These modifications depend on various factors including the recipient cell type, the source cell and the environmental stimuli received^15,24,25^. These effects are mediated by the molecular cargo, which is protected within EVs, thus keeping the integrity and functionality, particularly concerning microRNAs (miRNAs)^20^. The biocompatibility and the protection conferred by EVs to the cargo, allow these molecules to overcome biological barriers and adverse environments, such as the gastrointestinal tract, without damage, thus maintaining their functionality^26^. A study conducted in mice, reported that miRNAs loaded in mEV, can be detected in distant tissues after vesicle ingestion^27^.

Recently, milk has been particularly evaluated for the EV content due to the high vesicle amount and the promising applicability related to their multiple intrinsic functions^28,29^. Indeed, mEVs have already been investigated in many species (human, cow, donkey, goat, camel, buffalo, pig, and sheep)^30–35^ during different lactation periods, demonstrating an evolutionary conservation, especially for the RNA content. Several RNA types can be recognized in mEVs of different species, mainly miRNAs and mRNAs, but also other small RNAs (circular RNAs—circRNAs, Y-RNAs, long noncoding RNAs—lncRNAs, small nuclear RNAs—snRNAs, small nucleolar RNAs—snoRNAs, transfer RNAs—tRNAs, and piwi-interacting RNAs—piRNAs)^36–39^. In particular, four different miRNA families are often found among the most enriched in mEVs (cow, pig, human, goat, donkey and panda): miR-let-7, miR-30, miR-148 and miR-200^14,38^. Buffalo mEVs from mid-lactation milk have been previously evaluated by Chen et al.^14^, where 10 highly expressed miRNAs, accounting for three-quarters of all aligned reads, were referable to almost these last miRNA families.

Certainly, the role of miRNAs transported by animal mEVs in establishing cellular communication in the human receiver is still far from being fully understood, but more and more studies are reporting results that show the effect of mEV across different species and on many types of cells^40–42^. As a result, the anti-inflammatory and immunomodulatory properties of bovine mEVs were demonstrated in vitro in a 2D human model, reducing proinflammatory cytokine production and improving enterocyte homeostasis^43^. Similarly, goat mEVs improved swine enterocytes pre-stimulated with lipopolysaccharide (LPS), by reducing inflammation and increasing the expression of genes related to the mucosal barrier functions^44^. Moreover, different administration modes of bovine mEVs in mouse and pig in vivo models allowed the recovery of the labelled vesicles and the carried miRNAs in the liver, spleen, brain, heart and intestinal mucosa^45,46^. The effectiveness of this interspecies cross-talk mainly relies on the high conservation level in miRNA coding genes, which regulate numerous protein-coding genes in recipient cells, thus modifying several cellular processes^31,47–50^. In light of the aforementioned observations, this study aims to characterize the mEV small RNA content of Italian Mediterranean buffalo (*Bubalus bubalis*) milk during two unexplored lactation periods: colostrum of first milking and milk after fifty days of lactation. The objective is to compare data between periods and existing literature to evaluate whether these mEVs may harbor peculiar nucleic acids relevant for biological processes.

## Methods

### Colostrum and milk collection and extracellular vesicle isolation

From each of seven Italian Mediterranean buffalos, reared in five different farms located in central Italy (Lazio region), 500 ml from the whole milking of colostrum (first milking) and milk (fifty days after parturition) were obtained. Rearing conditions are standard intensive livestock systems. The seven subjects calved within 4 days range (from January 24th to January 28th, 2022). Individual colostrum and milk samples, from the same seven buffalos, were packaged in 50 ml plastic tubes containing Bronopol^®^ (2-bromo2nitropropano1,3diol) as a preservative, and immediately stored at 4 °C and processed within 24 h, avoiding cryo-preservation to minimize artifacts.

To isolate colostrum EVs (colosEVs) and mEVs, the protocol reported by Mecocci et al.^38^ was used. Colostrum and milk EVs were isolated through serial differential centrifugations (DC), and serum was treated with ethylenediaminetetraacetic acid tetrasodium salt dihydrate (EDTA) to precipitate most of the contaminating proteins. Fat globules and cellular debris and protein complexes were removed throw by applying preliminary DC steps: two sequential 3000 × g centrifugations for 10 min at room temperature (Eppendorf^®^ Centrifuge 5810R with a F34-6-38 rotor) and then a 10,000 × g for 1 h at 4 °C after the incubation for 15 min in ice with an equal volume of 0.25 M EDTA (pH 7.4). Afterward, ultracentrifugation at 35,000 × g for 1 h at 4 °C (Optima L-100 XP, Beckman Coulter, Milano, Italy) with a Type 45 Ti rotor (Beckman coulter) was carried out to eliminate precipitated proteins from sera, and a final ultracentrifugation at 200,000 × g for 90 min at 4 °C was applied to recover EV pellets.

### Colostrum and milk EV characterization

The effective isolation of colosEVs and mEVs was assessed by Transmission Electron Microscopy (TEM) observation and ExoView™ R100 technology (NanoView Biosciences, Brighton, MA, USA).

A drop of mEV suspension from one pellet was placed on Parafilm. A Formvar-coated copper grid (Electron Microscopy Sciences) was placed over each drop for approximately 30 min to allow the mEVs to adhere to the surface. The grids were then washed in PBS and distilled water and then contrasted with 2% uranyl acetate for 5 min. The samples were observed using a Philips EM208 transmission electron microscope equipped with a digital camera (University Centre of Electron and Fluorescence Microscopy—CUMEF). The ExoView™ R100 (NanoView Biosciences, Brighton, MA, USA) allowed us to test the isolated EVs for the positivity to EV markers such as cluster of differentiation 81 (CD81), CD9 and CD63, on a chip through an antibody-mediated capture. First, colosEV and mEV pellets were resuspended in 400 μl of 1 × PBS, filtered (0.22 μm pore size) and diluted 50 × in the incubation solution buffer of the human tetraspanin cargo kit (NanoView Biosciences, Brighton, MA, USA). Then, 50 μl of each solution were allowed to capture on the chip, left in incubation and washed, and finally made to react against the same fluorescently marked antibodies (CD81, CD9 and CD63). This technic also allowed to measure the size distribution and concentration of EV solutions.

### RNA extraction and library preparation

From each sample, four EV pellets were treated with 1 ml (each) of TRIzol™ (Thermo Fisher Scientific, Waltham, MA, USA) in order to extract total RNA content using the miRNeasy Mini Kit (QIAGEN, Germantown, MD, USA) and following the manufacturer’s instructions. On-column DNase digestion (RNase-Free DNase Set, QIAGEN, Germantown, MD, USA) was applied and the each extracted RNA (from 7 colosEV and 7 mEV samples) was quantified through the NanoDrop 2000 spectrophotometer (Thermo Fisher Scientific, Waltham, MA, USA) and quality tested by the Agilent 2100 Bioanalyzer RNA assay (Agilent technologies, Santa Clara, CA, USA). The checked RNAs (260/280 and 260/230 ratios and the RNA integrity number) were used to set up a small RNA library suitable for Next Generation Sequencing using QIAseq miRNA library kit (QIAGEN, Germantown, MD, USA) following the manufacturer’s instructions. Final libraries were checked with Agilent Bioanalyzer DNA assay and sequenced on a NextSeq 500 (Illumina, San Diego, CA, USA) system, generating paired-end 150 bp sequencies.

### Bioinformatic analysis

Raw reads from Illumina sequencer were checked for quality through the FastQC tool (https://www.bioinformatics.babraham.ac.uk/projects/fastqc/) and low quality/short reads and adaptors trimmed using TrimGalore (https://github.com/FelixKrueger/TrimGalore). BowTie2^51^ was used to align cleaned reads against reference features, setting parameters for short sequences. In particular, a two-step mapping procedure was carried out, first aligning on *Bos taurus* miRBase database (v.22) hairpin micro RNA (miRNA) sequences^52^ and then, the unmapped reads were used on *Bos taurus* reference genome (ARS-UCD1.2) to retrieve accurate information on miRNAs and other small RNAs. Unfortunately, we could not use the *Bubalus bubalis* genome and the specific miRBase since this species lacks annotation on miRNAs, which are the most represented features in the EV cargo. The uniquely mapped reads where selected and a comprehensive count matrix was built after the read counting for each feature type through FeatureCounts^53^ software, merging the information generated from the two alignments and summing counts for the same miRNA if covered by reads from both mapping steps. The normalized count matrix was generated through the DESeq2 package^54^ in R environment (v. 4.3.0)^55^ and used to evaluate feature abundances considering all genes with normalized counts greater than 1 in 4 out of 7 samples, for both colosEVs and mEVs.

An exploratory analysis was carried out on the same normalized count matrix by applying a principal component analysis on the top 100 features (*plotPCA* function of DESeq2), and a heatmap (*pheatmap* package). Finally, the differential gene expression analysis was carried out on genes with normalized counts greater than 1 in at least 7 out of 14 samples and considering as differentially expressed genes (DEGs) those with a False Discovery Rate (FDR) < 0.05 and a log_2_ Fold Change (FC) less than − 1 or greater than 1 (|log_2_ FC|> 1) in colosEVs compared to mEVs. The R package ggplot2^56^ was used to produce a volcano plot.

To better evaluate a possible sample specific message, miRNAs with a good expression level (comprising the 99.9% of total normalized counts) were kept, eliminating scarcely represented features, and specific (i.e. present in only one kind of sample) miRNAs of colosEVs (colosEV-specific miRNAs) and mEVs (mEV-specific miRNAs) were identified drawing a Venn diagram (https://bioinformatics.psb.ugent.be/webtools/Venn/). From this analysis a large core with miRNAs shared between the two conditions was highlighted and further refined by filtering for expression levels (top miRNAs comprising 95% of total normalized counts), in order to identify a set of miRNAs particularly enriched and shared between colosEVs and mEVs, ideally representing a buffalo specific signature (third column of Table 3).

### Functional analysis

The functional analysis was carried starting from the three lists of miRNAs already described colosEV-specific; mEV-specific and core miRNAs (shared by colosEV and mEV groups). Target genes were identified utilizing MiRWalk 3.0 (http://mirwalk.umm.uni-heidelberg.de/), with the input being the name of the most sequenced miRNA (5p or 3p) obtained from literature via miRBase (v.22). As a result, a list of target genes for each miRNA relative to the binding site on the targeted mRNA (3′-UTR, 5′-UTR, or CDS-coding region) was obtained. Then, for a more robust functional analysis, the targets were filtered for the number of miRNA hits, focusing on those genes targeted by about 50% of input miRNAs. This filter aims to identify the most probable targets likely to be modulated in receiving cells by colosEV or mEV upon uptake. Finally, the three lists of target genes derived from the initial groups of miRNAs were analysed with the Cytoscape 3.9.1 suite^57^. A Protein–Protein Interaction Network (PPI) was constructed based on the IMEx database^58^, and the network was clustered according to the number and type of connections between the nodes using the clusterMaker 2.0 app^59^ with the *gLay* option. With the most interconnected clusters (with 50 minimum number of interactions), a Gene Ontology (GO) enrichment analysis for biological process vocabulary was conducted, using the ClueGO application^60^. Results included only GO terms with a FDR < 0.05 (Benjamini Hockberg correction^61^). Moreover, to ease the reader's experience, a summary for the enriched GO terms for each cluster was provided, indicating functional group names within each cluster. Functional groups are identified by the ClueGO software based on related and interconnected enriched GO terms for a particular biological function and named with the most enriched term (lowest p-value).

### Data availability

We have submitted all relevant data of our experiments to the EV-TRACK knowledgebase (EV-TRACK ID: EV240033)^62^. The datasets containing raw sequence files of small RNA samples supporting the conclusions of this article are available in the Sequence Read Archive repository (SRA; BioProject ID PRJNA1091448—fourteen BioSamples from SAMN40597096 to SAMN40597109)^63^.

### Ethics approval and consent to participate

No approval of research ethics committees was required to accomplish the goals of this study because experimental work was carried out within the normal procedures for handling animals adopted by dairy buffalo farms hosting the trial. All applicable international, national, and/or institutional guidelines for the care and use of animals were followed. The procedure of colostrum and milk samples followed the routine procedure and out of the scope of Directive 2010/63/EU (art. 1.5.f “practices not likely to cause pain, suffering, distress or lasting harm equivalent to, or higher than, that caused by the introduction of a needle in accordance with good veterinary practice”).

## Results

### Extracellular vesicle evaluation

**Figure 1:**
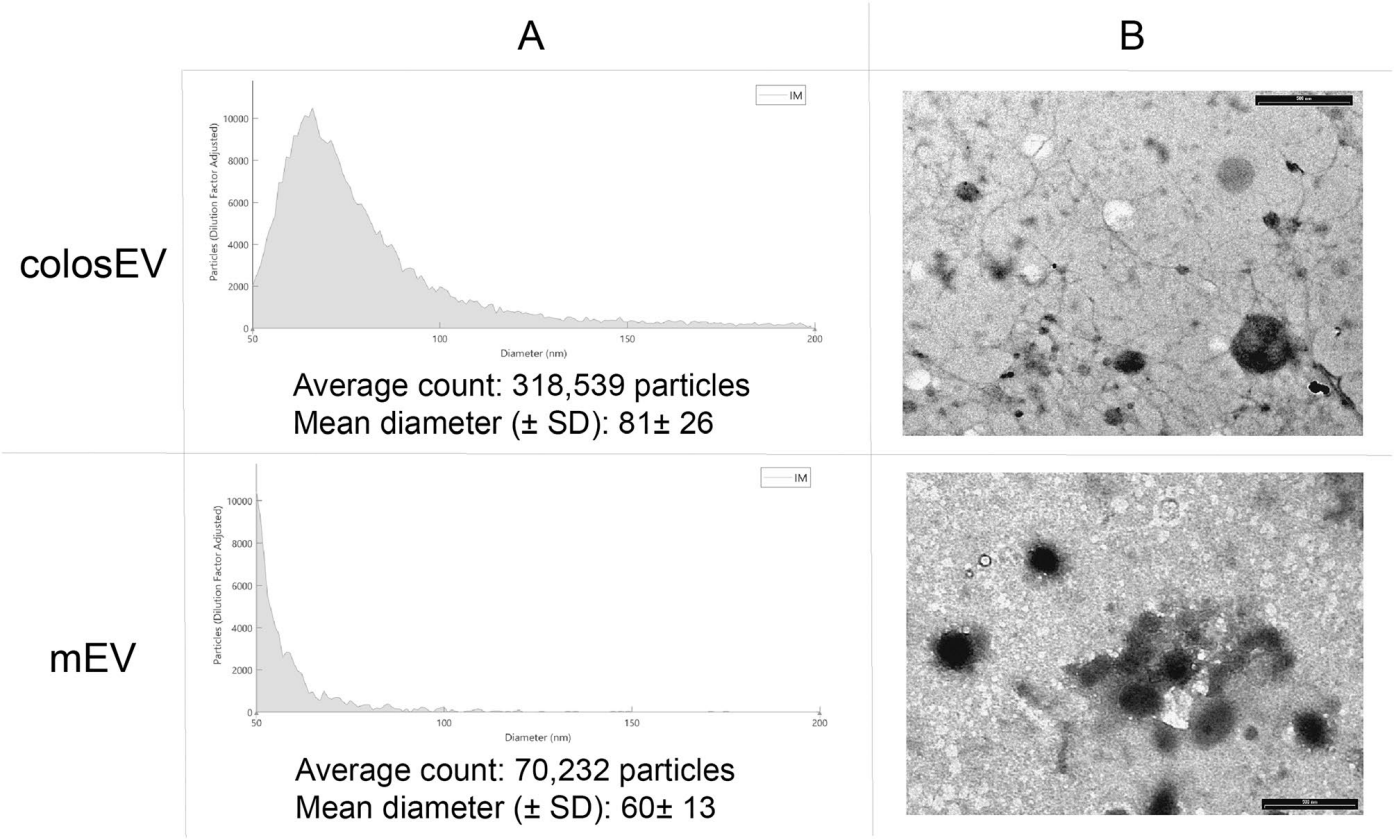
Morphological characterization of colostrum extracellular vesicles (colosEV) and milk extracellular vesicles (mEVs): (**A**) Exoview results obtained from the average of the analysis via interferometric imaging (IM) for the CD9 capture spot for colosEVsand mEVs; (**B**) Transmission electron microscopy (TEM) showing EVs enriched from colosEVsand mEV. Scale bar 500 nm.

The presence of EVs has been demonstrated through the Exoview technology. We observe a difference in terms of quantity and dimensions for milk and colostrum preparations. Indeed, from the anti-CD9 capture spot, colosEVs were about 4.5 times more concentrated with a slightly bigger mean diameter (Fig. 1A). All samples showed a higher cross-reactivity with the anti-CD9 antibody compared to anti-CD81 reaction, both detected by interferometric imaging. Differently, in fluorescence, the signal was specific and significant only for the anti-CD9 antibody. It was impossible to obtain data relating to anti-CD63 spots, as the results obtained were below the minimum detection limit, as expected, since no cross-reactions against this antibody were shown for mEVs in our previous characterizations^35,38^.

TEM analysis (Fig. 1B) revealed no specific differences between colosEVs and mEVs. The observed corpuscular elements were not particularly well preserved, but their morphology was consistent with typical EVs. They were between 40 and 300 nm in size, with most particles having a diameter of 100 nm or less. The field appeared to be fairly clean, apart from the presence of some filamentous structures, particularly in the colostrum.

### Sequencing output

Sequencing results for the seven colosEVs (C) and mEV (M) samples are reported in Tables 1 and 2 for first and second mapping, respectively. In detail, a deep sequencing was carried out with over 33 million raw reads for sample on average which become about 28 million after trimming procedures and quality check. After the first alignment step on miRNA database, about 16% of the cleaned reads were uniquely aligned (Table 1), in line with previous experiments on mEVs^38^.

**Table 1:** Sequencing statistics and alignment rate for the miRBase first mapping step. “C” colosEVs; “M” mEVs.

Sample	miRBase alignment						
	Raw reads	Trimmed input reads	Uniq	Uniq %	Multimapper	Multi %	Alignment rate
*B1_C*	45,090,492	38,406,997	6,336,291	16.50%	21,144,146	55.1%	71.6%
*B2_C*	23,835,663	19,523,663	3,160,046	16.19%	9,702,945	49.7%	65.9%
*B3_C*	33,186,245	24,342,794	4,070,186	16.72%	12,738,749	52.3%	69.1%
*B4_C*	25,621,270	21,459,235	3,650,778	17.01%	12,317,639	57.4%	74.4%
*B5_C*	28,445,463	25,598,326	4,822,819	18.84%	16,291,159	63.6%	82.5%
*B6_C*	35,297,007	28,794,068	5,851,514	20.32%	15,951,001	55.4%	75.7%
*B7_C*	27,625,173	21,786,905	4,316,988	19.81%	12,511,532	57.4%	77.2%
*B1_M*	46,868,375	37,915,680	6,077,164	16.03%	18,189,437	48.0%	64.0%
*B2_M*	42,131,819	36,397,508	4,570,048	12.56%	14,532,386	39.9%	52.5%
*B3_M*	32,171,579	26,518,421	4,078,482	15.38%	12,734,551	48.0%	63.4%
*B4_M*	38,699,411	32,599,923	4,189,562	12.85%	13,656,960	41.9%	54.7%
*B5_M*	25,322,872	21,707,379	2,779,265	12.8%	9,354,233	43.1%	55.9%
*B6_M*	38,190,192	30,878,366	4,678,766	15.15%	13,587,288	44,0%	59.2%
*B7_M*	31,895,400	25,705,878	3,692,451	14.36%	10,594,270	41.2%	55.6%
Mean	33,884,354	27,973,939	4,448,169	16.00%	13,807,593	49.8%	65.8%

**Table 2:** Alignment rate for second mapping step on the *Bos taurus* genome. “C” colostrum EVs; “M” milk EVs.

Sample	Genome alignment					
	Input reads (unmapped in the previous step)	Uniq	Uniq %	Multimapper	Multi %	Alignment rate
*B1_C*	10,926,560	2,175,921	19.91%	7,946,095	72.7%	92.6%
*B2_C*	6,660,672	937,680	14.08%	3,629,843	54.5%	68.6%
*B3_C*	7,533,859	1,364,108	18.11%	5,708,144	75.8%	93.9%
*B4_C*	5,490,818	1,046,342	19.06%	3,933,623	71.6%	90.7%
*B5_C*	4,484,348	928,592	20.71%	3,119,980	69.6%	90.3%
*B6_C*	6,991,553	1,395,589	19.96%	5,200,610	74.4%	94.4%
*B7_C*	4,958,385	1,111,651	22.42%	2,810,127	56.7%	79.1%
*B1_M*	13,649,079	2,158,686	15.82%	10,207,119	74.8%	90.6%
*B2_M*	17,295,074	2,251,371	13.02%	14,221,520	82.2%	95.3%
*B3_M*	9,705,388	1,942,881	20.02%	6,499,728	67.0%	87.0%
*B4_M*	14,753,401	2,146,013	14.55%	11,913,498	80.8%	95.3%
*B5_M*	9,573,881	1,101,063	11.5%	7,267,023	75.9%	87.4%
*B6_M*	12,612,312	1,929,848	15.3%	9,848,650	78.1%	93.4%
*B7_M*	11,419,157	1,602,801	14.04%	9,238,656	80.9%	94.9%
Mean	9,718,178	1,578,039	17.04%	7,253,187	72.5%	89.5%

About 1.6 million of the unmapped reads were identified in the second alignment step on the *Bos taurus* genome. Comparable results were obtained for the two sample types (Table 2).

### Small RNA cargo

**Figure 2:**
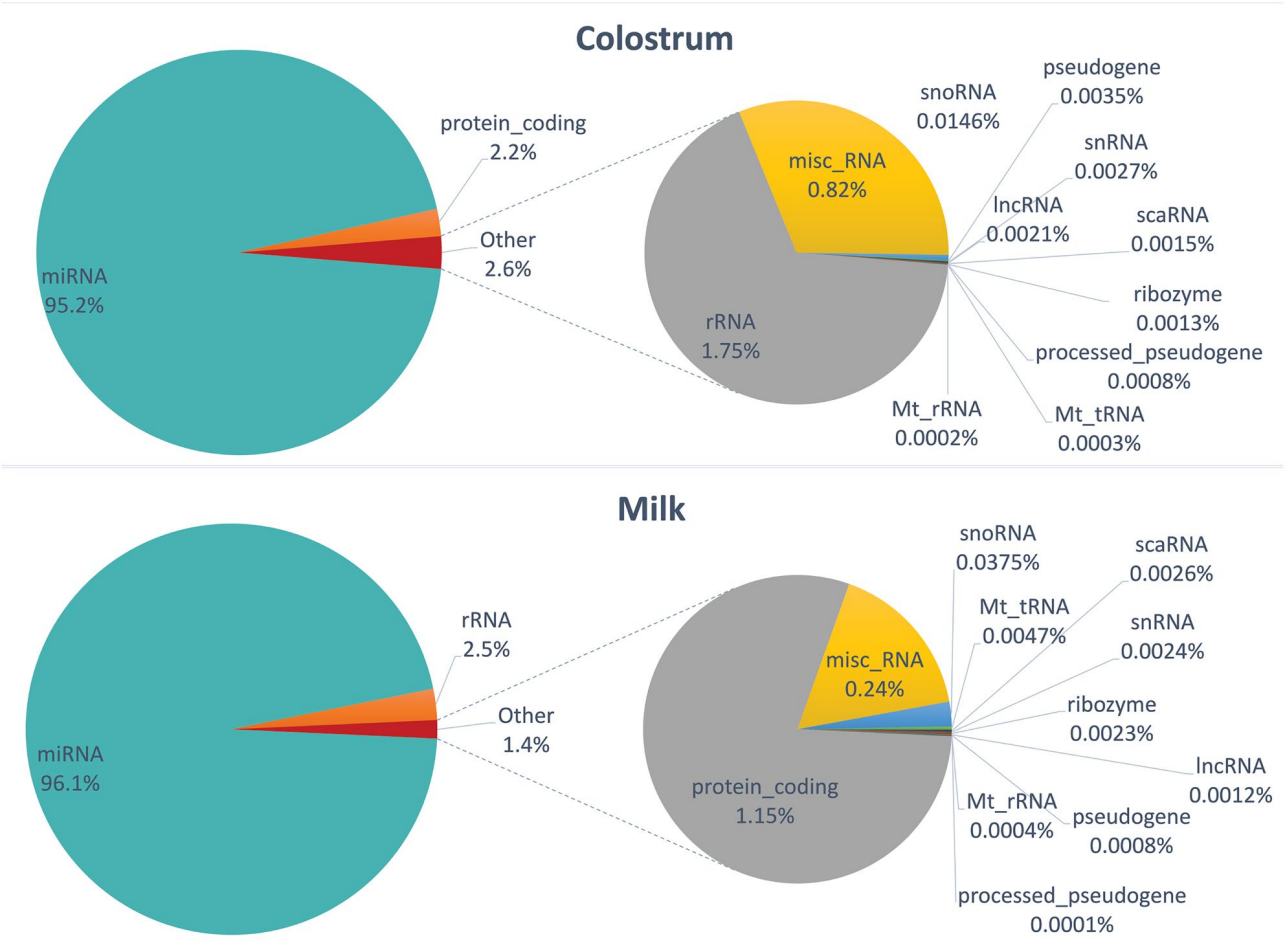
Small RNA abundances in colosEV and mEV cargos.

As expected from the alignment results and from previous studies^38,64^, most of the reads aligned to the miRNAs, included more than 95% of normalized counts for colosEVs and 96% for mEVs (Fig. 2).

**Figure 3:**
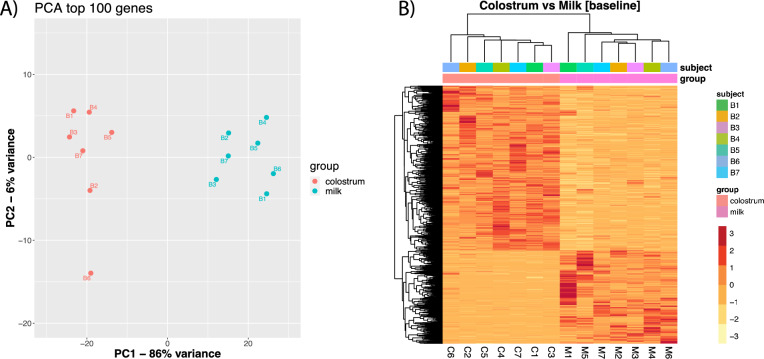
Exploratory analysis on small RNA normalized expression levels for buffalo EVs from colostrum (colosEV) and milk (mEV): (**A**) Results of the principal component analysis on first 100 features showing that 86% and 6% of the variance is explained by PC1 and PC2, respectively (red dots for colosEV and green dots for mEV samples); (**B**) heatmap of the seven subjects sequenced for small RNA cargo of colosEV and mEVs with samples in columns and features in rows.

A principal component analysis carried out on the count matrix shows a clear separation of the two groups along the first eigenvector (PC1), which explains 86% of the variance (Fig. 3A). This is confirmed by the heatmap analysis, which highlights two clearly defined clusters separated by sample type rather than individual animals (Fig. 3B).

**Figure 4:**
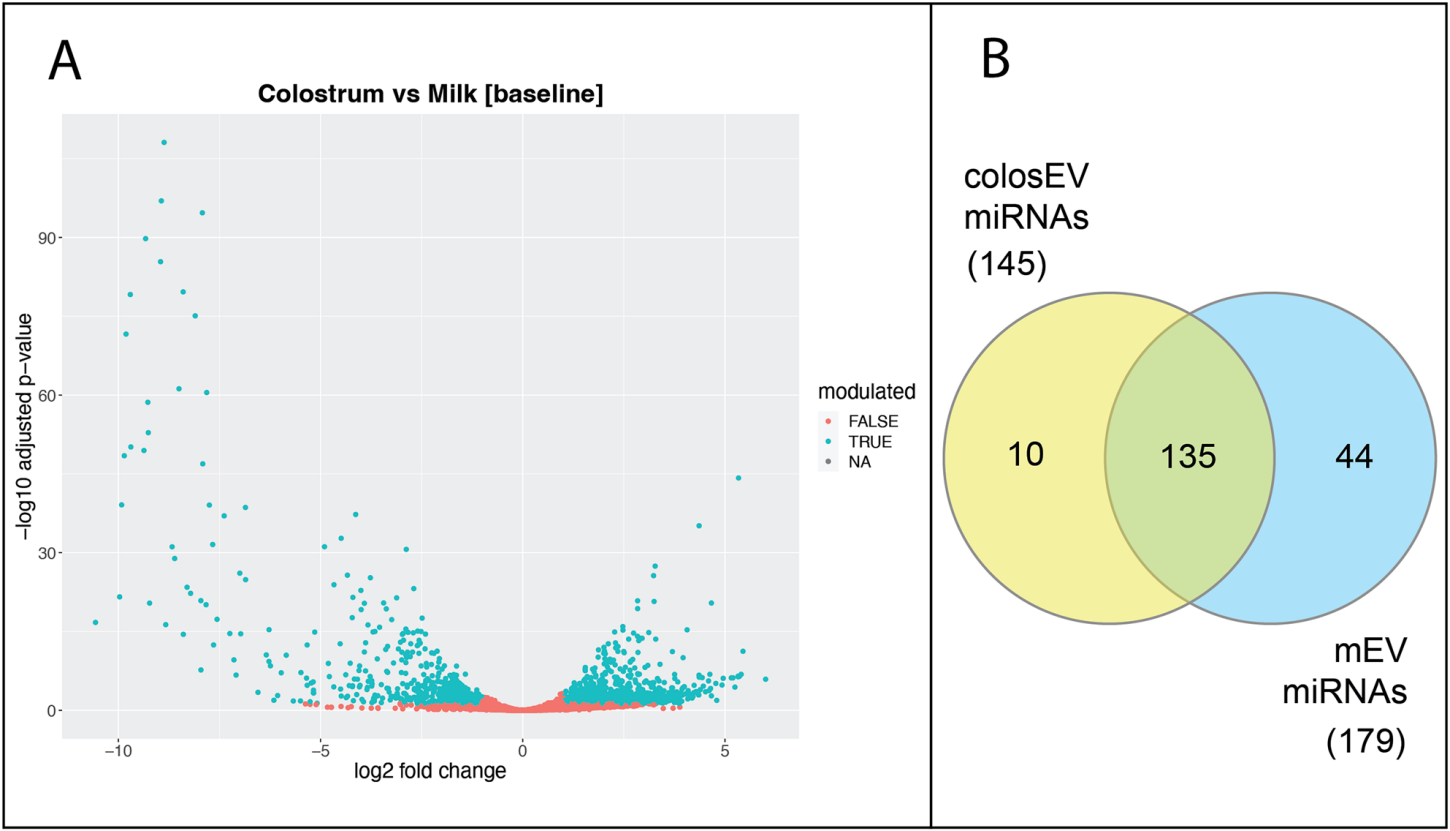
ColosEV and mEV small RNA cargo evaluation: (**A**) Volcano plot with differentially expressed genes (green dots, log2 Fold Change—log2FC >|1| and adjusted p < 0.05) in colosEVs vs mEVs; (**B**) number of miRNA including 99.9% of total normalized counts which are exclusively present in colosEVs (yellow) or mEVs (blue) or both (green).

This finding was further confirmed by the consistent number of differentially expressed features (Additional file 1). In detail, a total of 1504 differentially expressed genes (DEGs, log2 Fold Change − log2FC >|1| and adjusted p < 0.05) was found, 961 up-regulated and 543 down-regulated in colosEVs compared to mEVs (Fig. 4A; Additional file 1). Interestingly, an opposite scenario emerged for the predominant class miRNA: out of 212 differentially expressed features fewer (28) were up-regulated whereas a higher number of down-regulated (184) miRNAs in colosEVs compared to mEVs was found. Moreover, from the total miRNAs found in colosEVs (145) and mEVs (179), a consistent number was found expressed in a specific sample type (10 colosEV-specific and 44 mEV-specific) and completely absent (or with negligible counts) in the other type, as visible in the Venn diagram (Fig. 4B—first and second columns of Table 3).

**Table 3:** The restricted list of specific and shared miRNAs in colosEV and mEV cargos used for the functional analysis.

ColosEV-specific miRNAs	mEV-specific miRNAs	shared miRNAs
bta-mir-2284o; bta-mir-301a bta-mir-12048; bta-mir-204 bta-mir-133c; bta-mir-365-1 bta-mir-18b; bta-mir-211 bta-mir-2285cp; bta-mir-181d	bta-mir-103a-2; bta-mir-12031 bta-mir-487b; bta-mir-323 bta-mir-381; bta-mir-2285ap bta-mir-12038; bta-mir-12061 bta-mir-2889; bta-mir-299 bta-mir-543; bta-mir-154b bta-mir-3956; bta-mir-6516 bta-mir-12010; bta-mir-154a bta-mir-329b; bta-mir-92b bta-mir-485; bta-mir-412 bta-mir-453; bta-mir-493 bta-mir-495; bta-mir-380 bta-mir-411c; bta-mir-655 bta-mir-2284n; bta-mir-3578 bta-mir-136; bta-mir-758 bta-mir-323b; bta-mir-2285cl bta-mir-379; bta-mir-541 bta-mir-654; bta-mir-409a bta-mir-127; bta-mir-2892 bta-mir-2397; bta-mir-376e bta-mir-376d; bta-mir-154c bta-mir-665; bta-mir-1185	bta-mir-26b; bta-mir-423 bta-mir-185; bta-mir-3600 bta-mir-345; bta-mir-652 bta-mir-425; bta-mir-34a; bta-mir-29a; bta-mir-669 bta-mir-30a; bta-mir-181a-1 bta-mir-16a; bta-mir-125a bta-mir-499; bta-mir-191 bta-mir-660; bta-mir-362 bta-mir-200b; bta-mir-30d bta-mir-151a; bta-mir-200c bta-mir-375; bta-mir-30b bta-mir-15a; bta-mir-223 bta-mir-2285t; bta-mir-186 bta-mir-151

These were considered as sample type specific miRNAs and used for the functional analysis, in order to identify colostrum and milk specific messages. A great number of miRNA, however, was present in both EV types (135, Fig. 4B). From these, a restricted list comprising the most expressed miRNAs in both sample types was generated (Table 3, third column) and used for the functional analysis, to highlight main shared functions for colosEVs and mEVs. Mean and standard deviation values of normalized counts for miRNAs of each of the three lists are reported in Additional file 2.

### Functional analysis

The functional analysis was carried out as follows: targets were retrieved for each list of miRNAs (specific colosEVs, mEVs, and shared miRNAs), generating three lists of targets; for each one of the three lists of targets, a PPI-network was built and reorganized in clusters, identifying hub genes (central nodes); for each cluster, a functional analysis for enriched biological processes was carried out. In detail, for each miRNA list reported in Table 3, all the targets were retrieved and filtered on the number of miRNA hits, considering only those bound by 50% of input miRNAs (Table 4). Complete information for each target about binding miRNAs and binding site are reported in Additional file 3.

**Table 4:** Filtered targets based on the number of miRNA hits used for functional analysis.

Targets of specific colosEV miRNAs	Targets of specific mEV miRNAs	Targets of shared miRNAs
*DPF3; ZNF710; ZBTB37; WWC3; VDR; UBTF; SYNE3; SPSB1; SP1; RORA; PHF21A; PEAK1; OAZ2; NUDT3; MFHAS1; FBXO16; AMER2; C22H3orf49; LYST; ABCA7; USP9X; USP54; UNC80; MACF1; KMT2C; GYS1; DSCAM; DICER1*	*PRELID3A; RAB3C; NUDT3; LCOR; TFDP2; STRBP; SP1; PRLR; KIAA1147; FNDC10; ATG7; DNAH12; HMCN1; FAT2; SVEP1; PKHD1; DNAH9; DICER1; VCAN; USP34; PRKDC; PCSK5; LAMA1; CUBN; CDH23; APOB; TPP2; TG; SCN9A; PKHD1L1; LOC509283; LAMA3; HERC2; FAT4; AHCTF1*	*SLC24A4; PAG1; CXCL12; CLEC2B; NEB; LRP2; VPS13D; SACS; LOC509283; HECTD4; APOB; STARD9; PDE4DIP; NUP205; MYCBP2; HUWE1; CHD4; ACACA*

**Figure 5:**
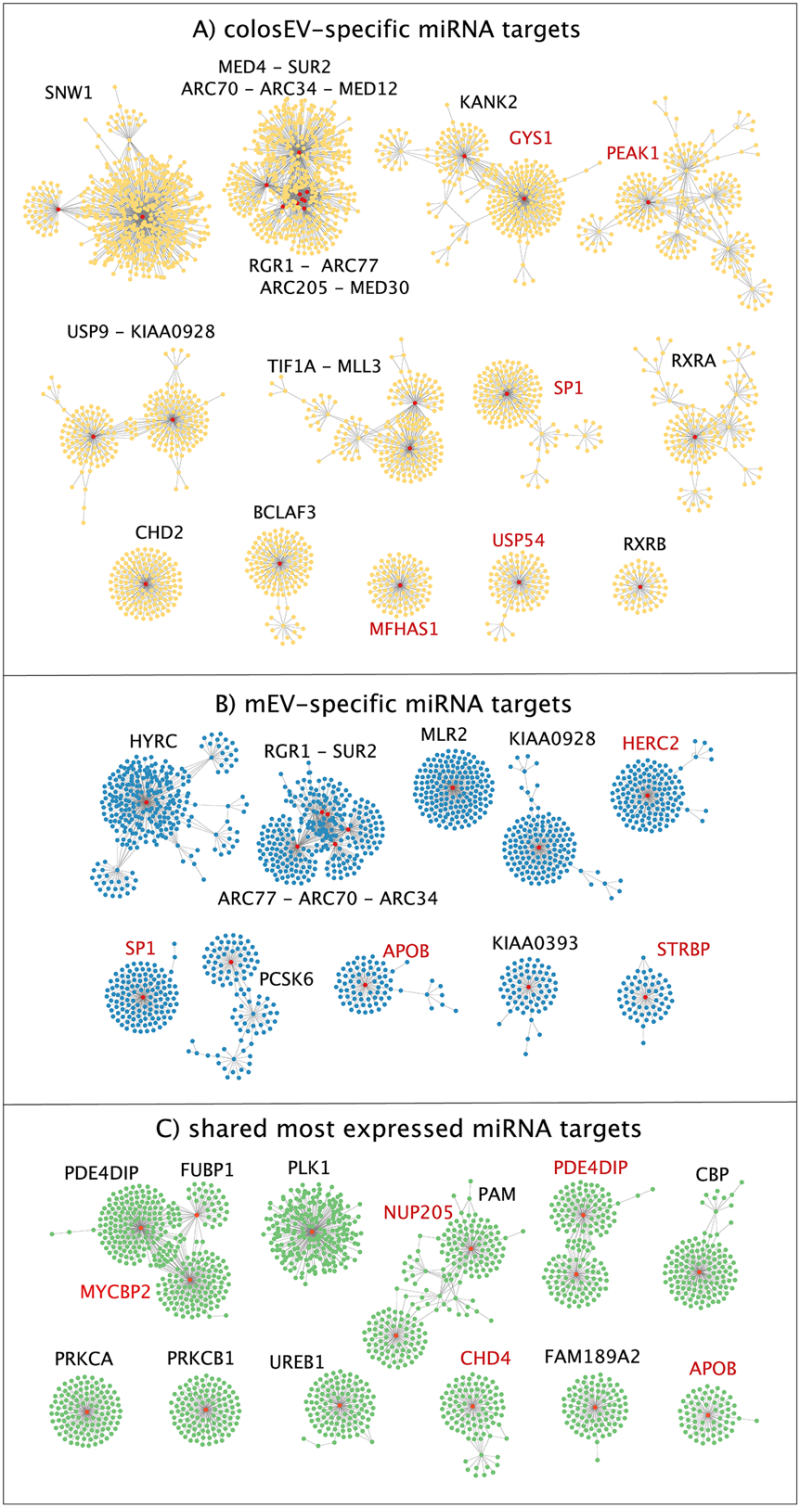
Clusters of protein–protein interaction (PPI)-network generated from targets of: (**A**) colosEV-specific miRNAs (yellow clusters); (**B**) mEV-specific miRNAs (blue clusters) and (**C**) shared miRNAs between colosEVs and mEVs (green clusters). Red nodes correspond to central nodes (proteins with more than 50 interactions) and relative protein names are reported close to each cluster (red names indicate miRNA targets). Clusters hereafter are named with progressive numbers as they appear in this figure and with the central node/nodes label.

The functional analysis was carried out starting from filtered targets, generating a Protein–Protein Interactions (PPI) network through the IMEx database. From the 28 targets of specific colosEV miRNAs, a complex network with 3275 nodes and 3918 edges was built, while for specific mEV miRNAs 2139 nodes and 2385 edges were found from the 35 targets. For the most expressed shared miRNAs, a network with 2483 nodes and 2564 edges was obtained from the 18 targets. These networks were separately organized in clusters based on the number of interactions between proteins, as reported in Fig. 5, resulting in 13, 10 and 11 clusters for colosEV-specific (Fig. 5A), mEV-specific (Fig. 5B) and core miRNA (Fig. 5C) targets, respectively.

A total of 24, 14 and 13 hub protein-coding genes, corresponding to proteins with the highest number of interactions with other proteins (over 50) sharing similar biological functions (red dots, Fig. 5), were found for colosEV, mEV and core miRNA targets, respectively. Two transcription factors, i.e. SP1 and TF1A, are among them but others are related to transcription such as the transcriptional cofactors ARC70, the coactivator SNW1, the coregulator BCLAF3 or the mediators ARC77, MED4, MED12, MED30, and RGR1. It is worth noting that some node proteins (ARC34, ARC70, ARC77, KIAA0928, RGR1, SP1 and SUR2) are shared between targets of specific colosEV and mEV miRNAs (Fig. 5A,B), while APOB is a central node of both mEV miRNA and core mRNA targets (Fig. 5B,C).

Moreover, some of the targets were also central nodes (whose names are written in red in Fig. 5): GYS1, MFHAS1, PEAK1, SP1, and USP54 for specific colosEV miRNAs; APOB, HERC2, SP1 and STRBP for specific mEV miRNAs; APOB, CHD4, MYCBP2, NUP205 and PDE4DIP for shared miRNAs.

For each of these clusters, a GO enrichment analysis for the “biological process” vocabulary was carried out through the ClueGO app and complete results are reported in Additional files 4–6 (if no data is reported, it means no enriched GO terms of functional group emerged). As a summary, Tables 5, 6 and 7 report the first 15 (lower adjusted p-value) functional groups in which are enclosed the enriched biological processes for each cluster. Functional groups are identified by the ClueGO software based on related and interconnected terms for a particular biological function among the enriched and are named as the most enriched comprise term (lower p-value).

**Table 5:** First 15 functional groups (lower adjusted p-value) of biological processes enriched for cluster of colosEV miRNA targets. For Clusters 11, 12 and 15, no statistically significant enriched terms were found.

colosEV miRNA targets	Function	Group adjusted p-value	no. of terms	Groups
*Cluster1* *SNW1*	RNA processing	1.0E−41	9	Group50
	Positive regulation of chromosome organization	5.8E−26	56	Group67
	DNA repair	4.8E−21	18	Group61
	Positive regulation of telomere maintenance	2.0E−19	40	Group65
	Positive regulation of DNA metabolic process	7.8E−19	35	Group64
	Regulation of DNA metabolic process	2.1E−18	10	Group53
	Regulation of chromosome organization	1.2E−17	53	Group66
	DNA metabolic process	1.2E−14	5	Group35
	Spliceosomal complex assembly	2.8E−14	3	Group27
	Cytoplasmic translation	1.2E−11	11	Group55
	Negative regulation of gene expression	3.0E−10	13	Group57
	Cell cycle checkpoint signaling	1.8E−09	13	Group58
	Regulation of RNA splicing	1.6E−08	8	Group46
	Mitotic sister chromatid segregation	2.1E−08	14	Group59
	Chromatin organization	2.1E−08	33	Group63
*Cluster2* *MED4-SUR2-ARC70-ARC34-MED12-ARG1-ARC77-ARC205-MED30*	Transcription initiation from RNA polymerase II promoter	2.6E−35	4	Group23
	Positive regulation of transcription initiation from RNA polymerase II promoter	3.5E−17	16	Group36
	Centrosome cycle	1.6E−12	27	Group38
	Histone acetyltransferase activity	6.1E−11	6	Group30
	Microtubule cytoskeleton organization	2.0E−09	12	Group32
	DNA-directed 5'-3' RNA polymerase activity	2.7E−08	2	Group17
	RNA polymerase II general transcription initiation factor activity	5.1E−08	19	Group37
	Histone H3 acetylation	9.8E−08	5	Group28
	Peptidyl-lysine modification	1.0E−07	3	Group19
	Intraciliary transport involved in cilium assembly	5.0E−07	13	Group35
	Regulation of RNA splicing	4.6E−06	3	Group20
	Nuclear receptor coactivator activity	5.3E−05	1	Group04
	Signal transduction in response to DNA damage	1.5E−04	13	Group34
	Nuclear mRNA surveillance	2.3E−04	13	Group33
	Intraciliary transport	2.3E−04	5	Group27
*Cluster3* *GYS1-KANK2*	Glycogen catabolic process	0.0E + 00	1	Group01
	Spinal cord motor neuron differentiation	0.0E + 00	1	Group03
	Vitamin D receptor signaling pathway	0.0E + 00	1	Group05
	Centriole replication	0.0E + 00	2	Group08
	Glycogen metabolic process	0.0E + 00	3	Group11
	Regulation of extrinsic apoptotic signaling pathway	0.0E + 00	3	Group12
	Cellular response to nitrogen starvation	0.0E + 00	5	Group13
	Negative regulation of G1/S transition of mitotic cell cycle	1.0E−02	1	Group07
	Positive regulation of cell-substrate adhesion	1.0E−02	2	Group09
	Regulation of glycoprotein biosynthetic process	2.0E−02	1	Group00
	Intermediate filament cytoskeleton organization	2.0E−02	1	Group04
	Negative regulation of cell migration involved in sprouting angiogenesis	2.0E−02	2	Group10
	Reciprocal meiotic recombination	3.0E−02	1	Group02
	Protein localization to microtubule cytoskeleton	3.0E−02	1	Group06
*Cluster4* *PEAK1*	Protein tyrosine kinase activity	4.4E−09	6	Group10
	Late endosome to lysosome transport	6.6E−06	16	Group11
	RNA polymerase II general transcription initiation factor activity	5.1E−05	4	Group09
	Positive regulation of protein localization to membrane	6.2E−04	2	Group08
	Hippocampus development	6.3E−04	1	Group01
	Regulation of potassium ion transmembrane transport	6.8E−04	2	Group06
	Regulation of early endosome to late endosome transport	7.5E−04	1	Group02
	Glutamate secretion	9.3E−04	1	Group00
	Regulation of long-term neuronal synaptic plasticity	9.3E−04	1	Group03
	axo-dendritic transport	2.5E−03	2	Group05
	Cellular response to amyloid-beta	2.8E−03	1	Group04
	Regulation of amyloid-beta formation	3.8E−03	2	Group07
*Cluster5* *USP9-KIAA0928*	Negative regulation of cellular amide metabolic process	3.9E−16	29	Group19
	Regulation of translation	2.0E−15	10	Group18
	Negative regulation of cellular macromolecule biosynthetic process	4.6E−15	7	Group15
	Regulation of mRNA stability	6.1E−15	6	Group13
	Regulation of mRNA metabolic process	4.4E−14	6	Group12
	mRNA destabilization	8.0E−14	8	Group16
	RIG-I signaling pathway	9.8E−10	20	Group17
	RNA transport	6.5E−09	6	Group14
	Peptidyl-serine phosphorylation	7.6E−09	1	Group05
	Protein tyrosine kinase activity	1.5E−07	3	Group07
	Regulation of cytokine-mediated signaling pathway	5.7E−07	4	Group10
	Regulation of type I interferon-mediated signaling pathway	5.1E−05	5	Group11
	Negative regulation of glycoprotein biosynthetic process	1.6E−04	1	Group04
	JUN kinase kinase kinase activity	1.8E−04	3	Group09
	Regulation of NIK/NF-kappaB signaling	2.9E−04	1	Group02
*Cluster6* *TIF1A-MLL3*	DNA-binding transcription activator activity. RNA polymerase II-specific	3.0E−20	1	Group00
	Chromatin organization	1.7E−12	1	Group04
	Mesenchymal cell apoptotic process involved in metanephros development	8.4E−12	37	Group14
	Gastrulation	8.7E−10	13	Group13
	Peptidyl-lysine modification	9.6E−10	3	Group09
	Regulation of kidney size	4.2E−08	13	Group12
	miRNA transcription	2.0E−06	3	Group08
	Camera-type eye morphogenesis	6.5E−06	4	Group11
	DNA methylation-dependent heterochromatin assembly	1.2E−04	4	Group10
	Cellular response to dopamine	1.4E−03	1	Group02
	Histone lysine demethylation	2.0E−03	1	Group01
	Protein sumoylation	2.3E−03	1	Group06
	Regulation of peptidyl-lysine acetylation	5.2E−03	1	Group03
	Centriole replication	5.3E−03	1	Group05
	Macrophage chemotaxis	6.7E−03	1	Group07
*Cluster7* *SP1*	miRNA transcription	7.0E−06	4	Group5
	Negative regulation of protein modification by small protein conjugation or removal	2.8E−04	1	Group1
	Fc-gamma receptor signaling pathway involved in phagocytosis	4.9E−04	1	Group3
	Regulation of cyclin-dependent protein serine/threonine kinase activity	5.0E−04	1	Group0
	Regulation of gene silencing by RNA	5.7E−04	3	Group4
	Endodermal cell differentiation	1.9E−03	1	Group2
*Cluster8* *RXRA*	Nuclear receptor activity	3.6E−07	1	Group0
	Regulation of myeloid cell differentiation	4.5E−07	7	Group5
	Cellular response to nutrient	1.6E−06	2	Group4
	Regulation of blood vessel endothelial cell migration	4.5E−05	1	Group1
	Positive regulation of keratinocyte differentiation	5.4E−05	1	Group2
	Regulation of microvillus assembly	8.2E−05	2	Group3
*Cluster9* *CHD2*	Maintenance of postsynaptic specialization structure	1.6E−05	2	Group3
	Intermediate filament cytoskeleton organization	2.7E−04	1	Group0
	Dendritic spine development	3.1E−04	1	Group2
	Regulation of focal adhesion assembly	6.4E−03	1	Group1
*Cluster10* *BCLAF3*	Acetyl-CoA biosynthetic process from pyruvate	0.0E + 00	1	Group0
	Centriole replication	0.0E + 00	1	Group1
	mRNA export from nucleus	0.0E + 00	10	Group2
*Cluster13* *RXRB*	Nuclear receptor activity	6.2E−10	1	Group0

**Table 6:** First 15 functional groups (lower adjusted p-value through Benjamini–Hochberg correction) of biological processes enriched for cluster of mEV miRNA targets. For Clusters 7, and 9–15, no statistically significant enriched terms were found.

mEV miRNA targets	Function	Group adjusted p-value	No. of terms	Groups
*Cluster1* *HYRC*	Blood vessel endothelial cell migration	4.20E−07	10	Group12
	Peptidyl-serine modification	4.80E−07	2	Group03
	Protein autophosphorylation	1.48E−06	4	Group10
	Autophagosome organization	1.81E−06	3	Group09
	Hematopoietic progenitor cell differentiation	2.03E−06	6	Group11
	Negative regulation of neuron death	2.49E−06	2	Group05
	Regulation of cardiac muscle hypertrophy	2.26E−04	2	Group02
	Cellular response to amyloid-beta	7.95E−04	1	Group01
	Positive regulation of transmembrane receptor protein serine/threonine kinase signaling pathway	8.44E−04	2	Group04
	Regulation of protein acetylation	1.49E−03	2	Group07
	Cellular response to angiotensin	4.32E−03	2	Group08
	Columnar/cuboidal epithelial cell differentiation	4.56E−03	2	Group06
	Regulation of T cell migration	8.40E−03	1	Group00
*Cluster2* *RGR1-SUR2-ARC77-ARC70-ARC34*	Positive regulation of transcription initiation from RNA polymerase II promoter	3.00E−40	8	Group20
	Transcription initiation from RNA polymerase II promoter	2.33E−35	15	Group23
	Transcription coregulator activity	6.63E−29	3	Group13
	RNA polymerase II general transcription initiation factor activity	1.14E−17	21	Group24
	Histone H3 acetylation	2.00E−11	4	Group17
	DNA-directed 5'-3' RNA polymerase activity	9.68E−11	2	Group10
	snRNA transcription by RNA polymerase II	6.01E−07	5	Group18
	Regulation of DNA recombination	5.89E−06	12	Group21
	Signal transduction by p53 class mediator	3.54E−05	13	Group22
	Regulation of telomere maintenance	1.60E−04	4	Group16
	Intracellular steroid hormone receptor signaling pathway	4.13E−04	2	Group12
	Positive regulation of attachment of mitotic spindle microtubules to kinetochore	1.45E−03	5	Group19
	Regulation of cyclin-dependent protein serine/threonine kinase activity	2.10E−03	2	Group08
	Maintenance of protein location in nucleus	2.68E−03	1	Group01
	Mitochondrial membrane organization	2.74E−03	4	Group15
*Cluster3* *MLR2*	Histone H3-K27 methylation	2.46E−06	9	Group3
	Positive regulation of amyloid precursor protein catabolic process	3.52E−04	3	Group1
	Low-density lipoprotein particle receptor activity	8.11E−04	1	Group0
	Positive regulation of establishment of protein localization to telomere	9.44E−04	9	Group2
*Cluster4* *KIAA0928*	mRNA metabolic process	3.68E−26	20	Group10
	Regulation of mRNA metabolic process	2.32E−25	11	Group08
	Regulation of cellular macromolecule biosynthetic process	5.24E−23	6	Group07
	Gene silencing by RNA	8.08E−21	16	Group09
	Negative regulation of cellular amide metabolic process	8.02E−20	3	Group04
	RNA transport	5.89E−12	6	Group06
	Translational initiation	1.16E−08	3	Group03
	Nuclear-transcribed mRNA catabolic process	2.04E−08	3	Group02
	Regulation of type I interferon-mediated signaling pathway	4.39E−05	3	Group05
	Positive regulation of mRNA splicing. via spliceosome	3.20E−04	1	Group00
	Spliceosomal snRNP assembly	9.07E−04	1	Group01
*Cluster5* *HERC2*	Translation initiation factor activity	2.77E−11	2	Group2
	Intermediate filament organization	7.01E−04	1	Group0
	Iron ion homeostasis	1.14E−03	1	Group1
	Negative regulation of TOR signaling	1.21E−03	3	Group3
*Cluster6* *SP1*	Cellular response to transforming growth factor beta stimulus	6.70E−09	7	Group5
	miRNA transcription	2.15E−06	2	Group3
	Negative regulation of protein modification by small protein conjugation or removal	4.94E−05	2	Group4
	Regulation of DNA-templated transcription in response to stress	1.94E−04	1	Group1
	Regulation of intracellular steroid hormone receptor signaling pathway	5.68E−04	1	Group0
	Cardiac muscle tissue morphogenesis	3.10E−03	1	Group2
*Cluster8* *APOB*	Regulation of polysaccharide biosynthetic process	6.94E−06	3	Group1
	Retrograde protein transport. ER to cytosol	2.10E−05	1	Group0

**Table 7:** First 15 functional groups (lower adjusted p-value through Benjamini–Hochberg correction) of biological processes enriched for cluster of shared miRNA targets. For Clusters 4, 5, 12, 13 and 15, no statistically significant enriched terms were found.

CORE miRNA target	Function	Group adjusted p-value	No. of terms	Groups
*Cluster1* *FUBP1-MYCBP2-PDE4DIP*	Actin cytoskeleton organization	5.74E−20	30	Group28
	Neuron projection morphogenesis	1.40E−18	12	Group25
	Regulation of plasma membrane bounded cell projection organization	8.12E−18	11	Group24
	Actin filament organization	1.21E−16	31	Group29
	Regulation of cytoskeleton organization	1.45E−16	17	Group27
	Cytoplasmic microtubule organization	1.58E−10	5	Group18
	GTPase regulator activity	3.30E−09	13	Group26
	Regulation of protein localization to membrane	1.07E−08	8	Group22
	Focal adhesion assembly	1.32E−07	9	Group23
	Regulation of cell projection assembly	3.11E−07	3	Group14
	Actin filament bundle organization	4.87E−07	7	Group21
	Protein localization to centrosome	2.77E−06	3	Group15
	Dendrite development	2.84E−06	3	Group13
	Dendrite morphogenesis	2.84E−06	5	Group19
	Microtubule polymerization	1.31E−05	6	Group20
*Cluster2* *PLK1*	Regulation of mitotic cell cycle phase transition	1.66E−09	31	Group13
	Mitotic nuclear division	9.31E−08	6	Group08
	Microtubule organizing center organization	4.49E−06	3	Group04
	Positive regulation of microtubule polymerization	4.58E−06	6	Group09
	Regulation of proteasomal protein catabolic process	4.78E−06	8	Group10
	Regulation of G2/M transition of mitotic cell cycle	9.71E−06	9	Group11
	5S class rRNA transcription by RNA polymerase III	9.97E−06	3	Group05
	Microtubule anchoring	1.01E−04	2	Group02
	Positive regulation of neuron projection arborization	4.67E−04	5	Group07
	DNA unwinding involved in DNA replication	6.94E−04	14	Group12
	Cellular response to ionizing radiation	1.32E−03	2	Group03
	Positive regulation of myeloid cell differentiation	2.92E−03	3	Group06
	Organelle transport along microtubule	2.97E−03	2	Group01
	Autophagosome assembly	3.09E−03	1	Group00
*Cluster3* *PAM-NUP205*	Nucleocytoplasmic transport	0.00E + 00	6	Group0
	Protein localization to microtubule cytoskeleton	0.00E + 00	1	Group1
	RNA transport	0.00E + 00	1	Group2
*Cluster6* *PRKCA*	5-Phosphoribose 1-diphosphate biosynthetic process	1.72E−08	1	Group0
	Tight junction assembly	7.50E−04	1	Group3
	Regulation of neurotransmitter secretion	1.07E−03	1	Group2
	Cellular response to amino acid starvation	1.52E−03	1	Group1
*Cluster7* *PRKCB1*	Nucleosome organization	1.21E−06	1	Group1
	Regulation of nuclease activity	2.70E−06	1	Group0
	Histone kinase activity	5.55E−05	1	Group2
	Regulation of protein dephosphorylation	5.78E−04	1	Group3
*Cluster8* *UREB1*	Protein deubiquitination	6.73E−06	1	Group0
	Protein K63-linked deubiquitination	8.05E−05	2	Group1
*Cluster9* *CHD4*	Chromatin remodeling	2.53E−16	1	Group0
	Histone deacetylation	9.88E−12	5	Group5
	ATP-dependent chromatin remodeler activity	4.70E−07	1	Group4
	Positive regulation of interleukin-1 production	2.58E−04	1	Group1
	Regulation of glial cell differentiation	2.94E−04	1	Group3
	Regulation of transcription from RNA polymerase II promoter in response to stress	7.26E−04	1	Group2
*Cluster10* *FAM189A2*	Translesion synthesis	7.71E−06	1	Group0
	Regulation of potassium ion transmembrane transport	3.49E−04	1	Group1
	Negative regulation of double-strand break repair	4.31E−04	1	Group2
*Cluster11* *APOB*	Retrograde protein transport. ER to cytosol	2.90E−05	1	Group0
	Regulation of polysaccharide biosynthetic process	3.69E−05	1	Group1

## Discussion

The findings of the present study on the small RNA content of buffalo colostrum and milk EVs might be interesting both for the development of the immune system and calf welfare. Moreover, they could shed light on the potential effect of buffalo milk could have on human nutrition. It is well known, indeed, that breast colostrum and milk have a key role in immune system enhancement and systemic disease resistance in infants; furthermore, breastfeeding is associated with reduction of morbidity and mortality^65^. Recently, strong immune regulatory functions have been recognized for the abundant immunoregulatory miRNAs in colosEVs and mEVs^64^. In particular, miRNAs are characterized by a strong evolutionary conservation across different animal species, leading to the possibility of interspecies cross-talk which potentially induces the regulation of several cellular processes^31,48–50^.

The first interesting result of the EV characterization was the one obtained through the ExoView™Assay, which represents an implementation of the Nanoparticle Tracking Analysis (NTA) to evaluate EV concentration and size by using specific EV antigens for vesicle detection. This method has an important advantage over NTA, allowing the measurement of only EVs and not of all the suspended nanoparticles. On the other hand, it can be challenging to find the right cross-reactivity against species-specific antigens, particularly in lesser-studied species such as buffalo. Nevertheless, it has been possible to have considerable EV detection with the anti-CD9 antibody. CD81, differently, showed a lower efficiency of capture and CD63 gave unreliable results, as already reported for cow mEVs^35,38^. As shown in Fig. 1, EVs were detected in both milk and colostrum preparations, although showing some differences in terms of quantity and dimension. As we expected, the colostrum was more concentrated in EVs than milk and was characterized by slightly larger vesicles. It is known that colostrum has considerable influence and importance for the newborn and is qualitatively different from milk. Indeed, colostrum not only possesses a greater quantity of proteins, mainly antibodies^12^, compared to milk, but also a different composition in EVs carrying their immunomodulatory message, as highlighted in this study.

Concerning the molecular cargo, the sequencing produced over 33 M raw sequences (reads) on average, a notably high number for small RNA investigations, which facilitate the detection of the great part of RNAs enclosed in colosEVs and mEVs. As expected from our previous study on milk-derived EV cargo characterization^38^, about 21.5% of quality-controlled reads are uniquely aligned to miRBase and genome, overall. Moreover, the paired sampling lowered the individual variability increasing the statistical power of data obtained. This allowed us to discern a distinct molecular cargo composition between colosEVs and mEVs as illustrated by the principal component analysis (PCA) and the heatmap (Fig. 3), which reflects the differences of the two matrices. Indeed, the principal component 1 (PC1) of panel A completely divides the two types of EVs, explaining 86% of total variability, and panel B shows two clear clusters deriving from the two sample matrices. It is worth noting that in this study, we obtained samples from the same seven animals in the two times. Hence, the PCA analysis of Fig. 3 also demonstrates that individual animals have minimal influence on the two profiles.

Most of the features carried by both colosEV and mEV cargos (about 95% of the total feature types) are represented by miRNAs and, due to their predominance and impact on the post-transcriptional gene expression regulation, we focused our attention on this class of RNAs, retrieving the most probable interested target genes and discussing related biological processes that can be affected in receiving cells once EVs are taken up. With an opposite trend compared to total DEGs, miRNAs showed to be more down-regulated (184) and less up-regulated (28) in colosEVs *vs* mEVs, identifying a sort of enrichment for miRNAs in mEV cargo. Moreover, we identified sample specific miRNAs, expressed in a sample type and completely absent (or with negligible counts) in the other (Venn diagram of Fig. 4B, Table 3). However, most of the miRNAs enclosed in colosEVs and mEVs are shared, in line with literature results on human milk^66^. In general, it is possible to identify some miRNA families particularly represented in both milk and colostrum EVs. Indeed, as for other species, bta-mir-30 and bta-mir-200 family members were found among the most abundant and shared between colosEVs and mEVs (Table 3) while a completely different result was obtained for other known miRNA families^29,67,68^. For instance, bta-mir-148 members were found in our buffalo colosEVs and mEVs although at concentrations under the threshold we applied to find the shared most expressed miRNAs, while for the let-7 family, generally hugely abundant in mEVs^38,69^, we found only bta-let-7f in both EVs at quite low concentrations.

Despite the apparent dilution of miRNAs within colosEV cargo, the functional analysis on their targets is characterized by a network with a much greater number of interactions than mEVs. Also, the number of hub genes from clusters and the enriched biological processes found statistically significant were greater for colosEVs than for mEVs, apparently indicating a higher degree of diverse actions associated with colostrum EVs (compare Tables 5 and 6, Additional file 4 and Additional file 5).

Interestingly, many GO terms related to the epigenetic regulation were found enriched in all the three lists of genes, although colosEV miRNA targets showed a higher number of enriched biological processes related to DNA metabolism and chromatin organization. Similar results were obtained for terms related to transcriptional and translational regulations including protein phosphorylation, being colosEV miRNA target list particularly interested by an enrichment of these processes.

Enriched GO terms related to cell cycle, cytoskeleton organization, vesicular transport and ion transport were found for targets of core shared miRNAs (Table 7 and Additional file 6) although further regarding colosEV miRNA targets. Interestingly, biological processes of neuronal development as well as protein kinase activity were found for all the three lists with tight-junction development found among core miRNAs. However, specific terms associated with cell differentiation and microvillus assembly were observed for colosEV miRNA targets. Concerning mEV miRNA targets, biological processes for cardiac and blood vessel development as well as mitochondria emerged.

Many biological processes related to the immune modulation were found for both colosEV and mEV samples: regulation of type I interferon and interleukin 1 (IL-1) production. Moreover, colosEV additionally showed “regulation of cytokine signalling pathway”, “regulation of NFKB signal”, “macrophage chemotaxis” and “FC-gamma receptor phagocytosis”, while “regulation of T cell migration” and “cell response to TGFB” were enriched biological processes for mEV miRNA targets.

Discussing more in detail the specific cargo message for colosEVs, we highlighted bta-mir-2284o, among the specific miRNAs, a typical bovine mammary gland miRNA known to be expressed in colostrum and decreased in abundance over time^70^. Also bta-mir-2285cp was exclusively found in colosEVs, which is a liver bovine miRNA that was predicted to regulate amino acids transportation by targeting solute carrier family 7, member 8 (*SLC7A8*) gene^71^. Another colostrum specific miRNA, reported to play a regulatory role in the immune system, is bta-mir-301a, for which an increased level was observed following T cell activation. This miRNA can activate "nuclear factor kappa-light-chain-enhancer of activated B cells" (NF-κB) signaling, increasing pro-inflammatory cytokines such as interleukin-8 (IL-8), interferon beta (IFN-β), nitric oxide synthase 2A (NOS2) and cytochrome oxidase subunit 2 (COX-2)^72^. Moreover, estrogen receptor α (*ERα*), one of the estrogen hormone-activated transcription factors, which regulates a large number of genes and is involved in the mammary gland development, is a target of miR-301a^73^. Mouse orthologue of bta-mir-204 can promote the synthesis of milk lipids in mammary epithelial cells by targeting *SIRT1*^74^. Moreover, in goat, mir-204 together with mir-211 regulates αS1-casein and β-casein synthesis via targeting αS1-casein coding gene in mammary epithelial cells^75^. In bovine, mir-204-5p may target several genes with roles in the nutritional regulation of gene expression in the mammary gland^76^. miR-365 in breast milk promotes the differentiation process of the brown adipose tissue through an expression improvement of the “Runt-related transcription factor 1, translocated to 1” (RUNX1T1) and induces, together with others, the inhibition of the cell proliferation, downregulating the expression of target genes in the p53 pathway^77^. Among specific colostrum miRNA, in a study aimed at investigating the potential regulatory role of miRNAs in the development of gastrointestinal tract, during the early life of dairy calves^78^, mir-211 was predicted to be related to gut epithelial cells and immune cell development, and to inflammatory response.

In the present study, from the 15 functional groups of biological processes enriched for clusters of colosEV miRNA, it has been highlighted processes linked to biosynthesis of macromolecules and regulation of immune signals as “negative regulation of cellular amide metabolic process”, “regulation of NIK/NF-kappaB signaling” for cluster 5 or “glycogen metabolic process” for cluster 3, “cellular response to nutrient” for cluster 8 and “macrophage chemotaxis” for cluster 6, as well as several GO terms related to signal transduction in clusters 4 and 5. Interestingly, for GO of upregulated miRNAs in mEV, and thus downregulated in coloEVs, enriched terms related to milk component synthesis were found for clusters 4, 5, 7 and 8, indicating the lack of repression of genes involved in these processes for colosEVs. Moreover, many transcription factors such as SP1 were found as central nodes for both colosEV and mEV miRNAs, indicating a great potential in gene expression regulation by miRNAs once EVs are taken up by recipient cells.

Moreover, the most interesting message that comes out from the biological processes enriched for cluster of colosEV miRNA targets pertain the epigenetic regulation: specifically, in cluster 1 we observed terms such “regulation of chromosome organization” and “DNA metabolic process”; in cluster 2 “histone acetyltransferase activity” and “histone H3 acetylation”; or in cluster 6 “chromatin organization” and “DNA methylation-dependent heterochromatin assembly”.

Hub of cluster 1, SNW1, is a protein previously shown to be involved in both splicing and transcription; its role involves binding to the NF-kappaB-p-TEFb complex to facilitate transcriptional elongation of some NF-kappaB target genes. Indeed, SNW1 complex has been identified as a novel transcriptional regulator of the NF-κB pathway^79^.

For cluster 2, several hubs (MED4, MED12, MED30, ARC70, ARC34, ARC77 and ARC205) are genes encoding for components of the mediator complex (MED) also known as activator-recruited cofactor. Mediator complexes are large multiprotein units that regulate gene expression in all eukaryotes and are involved in the transcriptional elongation and termination, mRNA processing, noncoding RNA activation, super enhancer formation, and epigenetic regulation^80^. MED consists of 31 subunits (MED1–MED31) in which MED12 is a critical transducer of regulatory information essential for organogenesis. MED4, instead, encodes for the vitamin D receptor-interacting protein (DRIP) complex, which works as a nuclear receptor coactivator essential for vitamin D metabolism. Moreover, recent studies linked MED4 to epigenetic regulation through a non-canonical pathway where MED4 depletion results in profound changes in the three-dimensional chromatin architecture in contrast to the canonical function of the Mediator complex^81^.

Nevertheless, hub genes of cluster 6 are TIF1A (transcription intermediary factor 1 α), known to interact with numerous proteins involved in chromatin structure^82^ and MLL3, a mono-methyltransferase that targets lysine 4 (Lys4) from histone 3 (H3K4), an epigenetic mark that has been related to enhancer elements involved in the activation of tumor suppressor genes^83^.

In conclusion, the whole message carried by the EVs from the two matrices (colostrum and milk) appears to be similar; the main difference is made by the amount since EVs are 10 to 100-fold higher in colostrum than in milk.

ColosEVs carry molecules, especially miRNAs, potentially capable of modifying metabolic processes of recipient cells involved in signal transduction, cell cycle and immune response, as for EVs of other previously characterized species, but with a special enrichment for miRNAs with epigenetic regulation capacities.

These beneficial characteristics of colosEVs are essential for the calf and could also be exploited for therapeutic purposes in humans, although further studies are necessary to measure the sanitization treatment impact on EV conservation, especially in buffalo where milk is consumed almost exclusively after processing.

### Supplementary Information

Supplementary Information 1.

Supplementary Information 2.

Supplementary Information 3.

Supplementary Information 4.

Supplementary Information 5.

## Data Availability

Supporting data and materials are submitted as Additional files and deposited in relevant repositories as indicated in materials and methods section.

## Abbreviations

EVs: Extracellular vesicles
mEVs: Milk extracellular vesicles
miRNA: MicroRNA
colosEVs: Colostrum EVs
GO: Gene ontology
MVBs: Multi-vesicular bodies
circRNAs: Circular RNAs
lncRNAs: Long noncoding RNAs
snRNAs: Small nuclear RNAs
snoRNAs: Small nucleolar RNAs
tRNAs: Transfer RNAs
piRNAs: Piwi-interacting RNAs
LPS: Lipopolysaccharide
EDTA: Ethylenediaminetetraacetic acid tetrasodium salt dihydrate
TEM: Transmission electron microscopy
CD81: Cluster of differentiation 81
CD63: Cluster of differentiation 63
CD9: Cluster of differentiation 9
FDR: False discovery rate
SRA: Sequence read archive repository
PPI: Protein–protein interactions
NTA: Nanoparticle tracking analysis
PCA: Principal component analysis
PC1: Principal component 1
*SLC7A8*: Solute carrier family 7, member 8
NF-κB: Nuclear factor kappa-light-chain-enhancer of activated B cells
IL-8: Interleukin-8
IFN-β: Interferon beta
NOS2: Nitric oxide synthase 2A
COX-2: Cytochrome oxidase subunit 2
*ER**α*: Estrogen receptor α
RUNX1T1: Runt-related transcription factor 1, translocated to 1
MED: Mediator complex
DRIP: Vitamin D receptor-interacting protein
TIF1A: Transcription intermediary factor 1 α
