# Supplementary material for: Comparison of colostrum and milk extracellular vesicles small RNA cargo in water buffalo
Source: Sci Rep. 2024 Aug 3;14:17991. doi: 10.1038/s41598-024-67249-6 (PMC11297979; doi:10.1038/s41598-024-67249-6)
Supplement: Supplementary file 2 — Supplementary Information 2. [file 41598_2024_67249_MOESM2_ESM.pdf]

Mean counts and standard deviation of the mean counts for each sample group of colosEV specific, mEV specific and shared miRNA reported in Table 3

List	miRNA	Counts (mean) colosEVs	Counts (SD) colosEVs	Counts (mean) mEVs	Counts (SD) mEVs
coloEV specific miRNAs	bta-mir-12048	13.5	3.8	3.3	2.0
	bta-mir-133c	11.9	11.4	1.1	2.5
	bta-mir-181d	16.2	17.0	4.9	3.4
	bta-mir-18b	12.8	7.4	4.7	4.9
	bta-mir-204	15.4	9.6	5.1	5.2
	bta-mir-211	10.7	11.5	2.1	3.1
	bta-mir-2284o	14.7	7.8	6.1	5.6
	bta-mir-2285cp	26.5	18.9	2.0	1.8
	bta-mir-301a	16.8	11.1	8.8	14.1
	bta-mir-365-1	7.8	5.4	2.7	4.0
mEV specific miRNAs	bta-mir-103a-2	4.6	2.0	15.6	11.0
	bta-mir-1185	1.4	1.9	143.9	114.1
	bta-mir-12010	4.1	2.2	33.0	17.4
	bta-mir-12031	5.2	2.6	28.2	40.4
	bta-mir-12038	3.0	3.6	16.2	9.0
	bta-mir-12061	6.1	3.8	38.9	16.7
	bta-mir-127	1.4	2.0	284.9	177.6
	bta-mir-136	0.2	0.4	27.5	27.0
	bta-mir-154a	1.0	0.8	280.5	202.4
	bta-mir-154b	4.0	6.4	1302.3	869.9
	bta-mir-154c	0.3	0.7	23.7	19.3
	bta-mir-2284n	3.2	3.0	86.6	39.0
	bta-mir-2285ap	1.3	1.3	39.7	21.2
	bta-mir-2285cl	0.0	0.0	21.0	12.2
	bta-mir-2397	5.7	2.7	18.6	10.1
	bta-mir-2889	2.9	2.7	17.4	13.9
	bta-mir-2892	2.6	1.5	24.8	15.7
	bta-mir-299	3.7	5.1	993.7	670.8
	bta-mir-323	3.1	2.7	1095.9	643.5
	bta-mir-323b	0.8	0.8	497.7	338.5
	bta-mir-329b	0.1	0.2	44.3	25.2
	bta-mir-3578	0.1	0.2	63.6	45.1
	bta-mir-376d	0.2	0.6	55.1	37.1
	bta-mir-376e	0.5	1.1	103.4	79.3
	bta-mir-379	5.2	5.8	2766.6	1714.2
	bta-mir-380	2.6	4.0	395.1	257.9
	bta-mir-381	0.1	0.2	18.5	14.1
	bta-mir-3956	0.0	0.0	58.7	52.9

	bta-mir-409a	0.5	1.4	346.9	206.7
	bta-mir-411c	2.3	2.9	1630.7	979.0
	bta-mir-412	0.1	0.4	36.3	24.5
	bta-mir-453	0.1	0.2	86.7	48.9
	bta-mir-485	0.5	0.8	175.1	137.4
	bta-mir-487b	3.3	3.7	1798.1	1225.2
	bta-mir-493	2.3	4.9	988.2	605.8
	bta-mir-495	0.0	0.0	23.1	17.4
	bta-mir-541	0.7	0.9	139.1	109.8
	bta-mir-543	0.1	0.2	227.6	153.7
	bta-mir-6516	3.9	2.6	38.8	23.8
	bta-mir-654	0.3	0.9	40.0	28.2
	bta-mir-655	0.2	0.5	115.8	78.3
	bta-mir-665	0.5	1.1	84.2	70.9
	bta-mir-758	0.5	1.0	34.7	31.3
	bta-mir-92b	2.9	2.8	19.9	8.5
shared miRNAs	bta-mir-125a	15323.4	7943.0	34360.4	13644.9
	bta-mir-151	186641.8	75996.3	621871.5	220765.2
	bta-mir-151a	6314.3	2686.3	17746.8	4912.5
	bta-mir-15a	5305.1	4042.0	19875.0	13706.3
	bta-mir-16a	8844.5	4199.6	21960.2	9113.6
	bta-mir-181a-1	16350.8	6392.7	32591.5	10131.4
	bta-mir-185	14064.0	5921.9	40379.9	15794.0
	bta-mir-186	24362.6	14147.7	72455.7	34403.4
	bta-mir-191	354367.4	120393.3	767216.1	255937.2
	bta-mir-200b	65246.2	33189.0	194652.8	67963.3
	bta-mir-200c	157425.1	69663.7	815732.9	337053.9
	bta-mir-223	1754.7	1989.4	23088.7	37910.7
	bta-mir-2285t	33096.8	18221.2	11339.9	9644.5
	bta-mir-26b	263461.1	122551.7	823206.4	473635.2
	bta-mir-29a	3148.6	1505.8	15188.6	8548.2
	bta-mir-30a	87689.0	34874.8	251887.4	106862.4
	bta-mir-30b	21239.9	14796.4	53941.5	33281.6
	bta-mir-30d	9528.0	3786.7	21751.6	8287.1
	bta-mir-345	4196.0	3027.4	9755.2	5692.8
	bta-mir-34a	63929.1	52377.3	9584.4	4024.0
	bta-mir-3600	3345.7	1517.8	48692.3	18087.4
	bta-mir-362	3161.8	1310.0	9897.9	3194.1
	bta-mir-375	2676.3	2703.4	419538.7	80000.7
	bta-mir-423	27727.6	15720.9	61562.8	28395.8
	bta-mir-425	9953.6	4310.8	22905.7	6275.5
	bta-mir-499	3015.8	2062.8	16312.9	12200.0
	bta-mir-652	7386.9	2981.6	17837.6	7288.5

	bta-mir-660	30289.0	14030.5	65301.5	27046.6
	bta-mir-669	4667.4	1676.4	33716.8	13363.6
